# Bioactive Composition and Free Radical Scavenging Activity of Fresh Orange Maize Hybrids: Impacts of Genotype, Maturity Stages, and Processing Methods

**DOI:** 10.3389/fnut.2021.640563

**Published:** 2021-02-24

**Authors:** Emmanuel Oladeji Alamu, Bussie Maziya-Dixon, Abebe Menkir, Emmanuel Anyachukwu Irondi, Olorunfemi Olaofe

**Affiliations:** ^1^International Institute of Tropical Agriculture, Southern Africa Research and Administration Hub (SARAH) Campus, Lusaka, Zambia; ^2^Food and Nutrition Sciences Laboratory, International Institute of Tropical Agriculture (IITA), Ibadan, Nigeria; ^3^Maize Breeding Unit, International Institute of Tropical Agriculture (IITA), Ibadan, Nigeria; ^4^Department of Medical Biochemistry and Pharmacology Kwara State University, Ilorin, Nigeria; ^5^Department of Chemistry, Ekiti State University, Ado Ekiti, Nigeria

**Keywords:** bioactive constituents, free radicals, maturity stages, orange maize, processing methods, hybrids, maize husks, Dehusking

## Abstract

Bioactive compounds in foods are responsible for their biological activities, but biotic and abiotic factors may influence their levels. This study evaluated the impact of three genotypes (designated 4, 5, and 7), maturity stages (20, 27, and 34 days after pollination) and processing methods (hydrothermal and dry-heating) on the bioactive constituents (carotenoids, phytate, tannins, vitamin C) and 2,2-diphenyl-2-picrylhydrazyl radical (DPPH^*^) scavenging activity of fresh orange maize hybrids. Freshly harvested maize cobs of each genotype were subjected to hydrothermal processing at 100°C and dry-heating with husks and without husks. Carotenoids (lutein, zeaxanthin, β-cryptoxanthin, α-carotene, and total β-carotene) contents of fresh and processed samples were analyzed using HPLC; other bioactive constituents and DPPH^*^ scavenging ability were determined using spectrophotometric methods. Genotype had a significant effect on the levels of carotenoids (*p* < 0.001) and vitamin C (*p* < 0.05), while genotype (*p* < 0.001), and processing methods (*p* < 0.001) had significant effects on DPPH^*^ SC_50_. Maturity stages, processing methods and their interaction also had significant effects (*p* < 0.001) on the levels of all the bioactive constituents. A positive moderate to strong correlation was observed between (*p* < 0.001) α-carotene and the following: lutein (*r* = 0.57), β-cryptoxanthin (*r* = 0.69), total β-carotene (*r* = 0.62). However, the relationship between α-carotene and zeaxanthin was positive but weak (*r* = 0.39). A positive moderate correlation (*p* < 0.001) was observed between lutein and the following: β-cryptoxanthin (*r* = 0.57), total β-carotene (*r* = 0.58), and zeaxanthin (*r* = 0.52). A positive strong correlation (*p* < 0.001) was observed between β-cryptoxanthin and each of total β-carotene (*r* = 0.92) and zeaxanthin (*r* = 0.63); total β-carotene and zeaxanthin (*r* = 0.65); while the association between vitamin C and DPPH^*^ SC_50_ was negative and weak (*r* = −0.38). Generally, genotype 4 and harvesting at 34 days after pollination had the best combination of bioactive constituents and DPPH^*^ scavenging ability.

## Introduction

Pigmented maize (*Zea mays* L.) grains are rich dietary sources of bioactive compounds, such as anthocyanins, carotenoids, polyphenolic compounds, vitamin C, and phytate (1, 2). These bioactive compounds are notable for their antioxidant activities, among other health benefits such as anti-obesity, antidiabetic, and anti-cancer activities (3–5). Antioxidant phytochemicals protect the cells against reactive oxygen species (ROS) and free radicals (6), and the oxidative damage they inflict on biomolecules in the cell, including proteins, lipids, and nucleic acids (7).

ROS and other oxygen free radicals are produced in the human body as by-products of many physiological and biochemical processes, especially, aerobic metabolism. Typically, endogenous antioxidant molecules, such as glutathione and arginine, and enzymes, such as catalase, superoxide dismutase, glutathione peroxidase and glutathione reductase, are available in the cell to scavenge the ROS and free radicals, thereby protecting the cells from oxidative damage (8). However, a preponderance of ROS and free radicals over the available antioxidant molecules in the cells results in oxidative stress, and oxidative stress-associated chronic diseases (9, 10). In addition to damaging cellular biomolecules and causing diseases, ROS and free radicals cause oxidative degradation of nutrients in food products (11). One of the approaches to boost the antioxidant status of the body is by increasing the intake of whole cereal grains and many other dietary sources of antioxidant compounds. This is because dietary antioxidants can end the chain reactions of free radicals through proton donation. In this context, orange maize has been reported to exhibit antioxidant activity, a property attributed to its bioactive compounds (2).

The levels of bioactive compounds and antioxidant activity of maize and maize products can be affected by biotic factors such as genotype and maturity stages, and abiotic factors such as processing methods and storage conditions (2, 12, 13). Furthermore, a recent study concluded that the retention of bioactive compounds in open-pollinated biofortified maize varieties during boiling (with and without husks) was genotype-dependent (1). However, there is an inadequacy of scientific information on the individual or combined effects of genotype, maturity stages, and processing methods on the levels of bioactive compounds and free radical scavenging ability of orange maize hybrids. Therefore, this study investigated the impact of genotype, maturity stages, and processing methods on the bioactive constituents and 1, 1–diphenyl-2-picrylhydrazyl free radical (DPPH^*^) scavenging activity of orange maize hybrids.

## Materials and Methods

### Materials

Cobs of three genotypes (designated 4, 5, and 7) of orange maize hybrids used for this study were freshly harvested from the Maize Improvement Programme research field of the International Institute of Tropical Agriculture (IITA), Ibadan, Nigeria. The orange maize hybrids were grown in Ibadan (7° 22 N, 3° 58 E; 150 m a.s.l.) in a randomized complete block design in three replicates. The cobs were harvested at maturity stages of 20, 27, and 34 days after pollination, which coincided with 57 days after planting, characterized by 50% anthesis/pollen shed or 50% silk emergence. A total of 90 cobs of each genotype were harvested at 08.00 h in each of the maturity stages. The cobs were immediately taken to the laboratory in mailing sacks and subjected to processing within 24 h after harvest (1).

Analytical grades of all chemicals and reagents were used for samples analyses.

### Processing of Fresh Orange Maize Hybrids Cobs

The cobs of each genotype of orange maize hybrids were randomly divided into five portions of 18 cobs each. The first portion (fresh/unprocessed) was not subjected to any processing, while each of the remaining four portions was subjected to one of the following processing methods: hydrothermal processing with the husks intact (HTH) and without the husks (HTWH); and dry-heating with the husks intact (DHH) and without the husks (DHWH). Dehusking of the maize cobs was carried out manually. Hydrothermal processing of maize cobs was carried out in stainless pots containing 2 L of tap water at 100°C, using domestic gas cooker (1, 14). The cooking time of cobs differed according to the maturity stage at which they were harvested. Cobs without husks were cooked for 25, 35, and 45 min, while those with husks were cooked for 35, 45, and 55 min for the maturity stages of 20, 27, and 34 days after pollination, respectively.

Dry-heating processing was conducted by placing the freshly harvested orange maize cobs with and without husks on wire gauze above burning charcoal until the seeds were done in line with the traditional practice described by Osanyintola et al. (14). Similar to the hydrothermal processing, the dry-heating processing time varied with the maturity stage at which the maize cobs were harvested, as earlier described by Alamu et al. (12) as follows. Cobs without husks from 20, 27, and 34 days after pollination were dry-heated for 15, 12, and 10 min, respectively; while cobs with intact husks from 20, 27, and 34 days after pollination were dry-heated for 20, 15, and 10 min, respectively.

Subsequently, the cobs in each sample portion were manually threshed, and the grains were lyophilized using Labconco Freezone 4.5 L lyophilizer at −54°C and 0.45 mbar. The lyophilized grains were milled into flour (0.5 mm particle size) and packed in polythene sample bags (whirl-pack). Samples for carotenoids profile were stored at −80°C, while those for other analyses were stored at 4°C during analyses.

### Quantification of Carotenoids

Carotenoids were quantified using HPLC system as per the method described by Howe and Tanumihardjo (15), with a slight modification (12). The HPLC system (Water Corporation, Milford, MA) comprised a C30 YMC carotenoid guard-column (4.6 × 250 mm, 3 μm), binary pump (Waters 626), auto-sampler (Waters 717), and photodiode array detector (Waters 2,996). Carotenoids were extracted from sample (0.6 g) with 6 mL of ethanol containing 0.1% butylated hydroxyl toluene in a water bath at 85°C for 5 min. Next, interfering oil in the mixture was saponified by adding 500 μL of potassium hydroxide (80% w/v), and heating twice at 85°C for 5 min in a water bath. The sample was transferred into an ice bath, and cold de-ionized water (3 mL) was added to it. After that, carotenoids were separated by mixing with 3 mL of hexane and centrifuging at 1,200 g for 5 min, for three consecutive times. The hexane fractions were pooled and washed thrice with de-ionized water, mixed and centrifuged at 1,200 g for 5 min. Hexane was vaporized in a vacuum rotary evaporator, and the carotenoids residue was reconstituted with 1 mL of methanol/dichloromethane (50:50 v/v), out of which 100 μL was injected into the HPLC system. The mobile phase was composed of solvent A [methanol:water (92:8 v/v), containing ammonium acetate (10 mmol/L)] and solvent B (100% methyl tertiary-butyl ether). Gradient elution programme was set at 1 mL/min at the following conditions: linear gradient from 83 to 59% A for 29 min; linear gradient from 59 to 30% A for 6 min; 1 min hold at 30% A; linear gradient from 30 to 83% A for 4 min; and 4 min hold at 83%. Identification and quantification of individual carotenoids were carried out at 450 nm by comparing their spectra and retention times with those of reference standards.

### Determination of Vitamin C Content

Vitamin C content of samples was determined using the titration method of AOAC (16). Sample (50 g) was homogenized for 3 min in 150 mL of an extraction solution containing aqueous metaphosphoric acid (3%) and acetic acid (8%). The resulting slurry was made up to 200 mL with the extraction solution, and vacuum-filtered using a Buchner funnel and Whatman filter paper (no. 1) to give the extract. After that, 50 mL of the extract was rapidly titrated with a standardized 2, 6-dichloroindophenol dye solution until a rose-pink color persisted for more than 5 s. The vitamin C content was subsequently calculated using the following formula:

Vitamin C (mg/100 g) = Titer value × 0.606 × 100/Weight of sample

### Determination of Phytate Content

Phytate content of samples was determined as per the method of Wheeler and Ferrel (17). Briefly, 5 g of sample flour was extracted with 50 mL of 3% TCA by mechanically shaking the mixture for 30 min and centrifuging at 3,500 rpm for 15 min. A mixture of 10 mL of the supernatant and 4 mL of FeCl_3_ solution (2 mg FeCl_3_ per mL of 3% TCA) was heated in a boiling water bath for 45 min. The suspension was centrifuged at 3,500 rpm for 15 min, and the precipitate was washed twice by dispersing in 25 mL of 3% TCA, heating in a boiling water bath for 5 min and centrifuging. The washing was repeated once with de-ionized water, after which the precipitate was dispersed in 3 mL of 1.5 M NaOH. After that, the volume was made up to 30 mL with de-ionized water, and the mixture was heated in a boiling water bath for 30 min. The suspension was filtered hot, and the precipitate was washed with 60 mL of hot water. The filtrate was discarded, and the precipitate from the filter paper was dissolved with 40 mL of hot 3.2 M HNO_3_ into a 100 mL volumetric flask. Subsequently, the filter paper was washed with several portions of de-ionized water, collecting the washings into the same flask and diluting to 100 mL. A 5 mL aliquot, diluted to 70 mL in another 100 mL volumetric flask, was mixed with 20 mL of 1.5 M KSCN and the volume was made up to 100 mL. Absorbance was read at 480 nm within 1 min against a reagent blank. The iron content was calculated from Fe(NO_3_)_3_ standard curve, and the phytate phosphorus was calculated from the iron content assuming a 4:6 iron:phosphorus molecular ratio.

### Determination of Tannins Content

Tannins content of samples was determined as per the method of Joslyn (18). Sample (0.75 g) was extracted with 20 mL of 50% methanol in a water bath at 77–80°C for 1 hr. The extract was filtered and made up to 50 mL, using 50% aqueous methanol. Afterwards, 1 mL of the extract was mixed with 20 mL of distilled water, 2.5 mL of Folin-Dennis reagent and 10 mL of 17% Na_2_CO_3_. The mixture was diluted to 50 mL with distilled water and incubated in the dark at room temperature for 20 min. Absorbance was read at 760 nm, and tannins content was calculated using a tannic acid standard curve.

### DPPH^*^ Scavenging Assay

DPPH^*^ scavenging assay was carried out according to the method reported by Chan et al. (19), using vitamin C as a reference antioxidant. Sample (0.5 g) was extracted with 25 mL of methanol by continuously shaking for 1 h at room temperature. Thereafter, the mixture was centrifuged at 3,000 rpm for 10 min, and the supernatant (extract) was collected. A portion of 2 mL of DPPH^*^ solution (5.9 mg/100 mL methanol) was mixed with varying dilutions of the extract, amounting to 1 mL. The mixture was incubated in the dark for 30 min at room temperature, after which the absorbance was read at 517 nm. Afterwards, the DPPH^*^ scavenging ability of the extract was calculated and expressed in terms of SC_50_ (that is, the concentration of extract that scavenged DPPH^*^ by 50%).

### Data Analysis

Results (non-transformed data) of triplicate analyses were subjected to descriptive statistics and analysis of variance (two-way ANOVA for the multivariate) using the Statistical Analysis System (SAS) software package. Least significant difference (LSD) test was used for mean comparison. Pearson correlation among the bioactive constituents levels and DPPH^*^ SC_50_ were calculated.

## Results and Discussion

### Carotenoids Profile of Orange Maize Hybrids as Affected by Genotype, Maturity Stages, and Processing Methods

Carotenoids profile (μg/g) of the orange maize hybrids as affected by genotype, maturity stages, and processing methods is presented in Table 1. The major carotenoids in the maize samples were lutein (1.27–7.59 μg/g DW), zeaxanthin (4.89–26.71 μg/g DW), β-cryptoxanthin (0.96–7.01 μg/g DW), α-carotene (0.09–1.18 μg/g DW), and total β-carotene (1.11–6.08 μg/g DW). The presence of these carotenoids in orange maize genotypes was reported by previous studies (1, 20, 21). Generally, the levels of xanthophylls (lutein and zeaxanthin) were higher than those of provitamin A carotenoids (β-cryptoxanthin, α-carotene, and β-carotene) in the orange maize hybrids, as reported by previous studies (22, 23), with zeaxanthin as the predominant carotenoid (22, 24). Provitamin A carotenoids-biofortified orange maize genotypes are prominent vehicle for the alleviation of vitamin A deficiency, especially in developing countries (20, 21). On the other hand, xanthophylls are well-known for the important role they play in maintaining good eye health and in reducing the risk for age-related macular degeneration, which is the major cause of blindness in older people (13, 25). Carotenoids are also well-known for their antioxidant activities and protective effects against chronic diseases such as cardiovascular diseases (26) and cancer (27); with their antioxidant activity being the main mechanism of their health benefits (28). Thus, the presence of both provitamin A carotenoids and xanthophylls in the orange maize hybrids in this study suggests that their intake may be beneficial for the prevention and/or alleviation of vitamin A deficiency and oxidative stress.

**Table 1:** Carotenoids profile (μg/g dry weight) of orange maize hybrids as affected by genotype, maturity stages, and processing methods.

**Summary**	**Lutein**	**ZXT**	**BCX**	**AC**	**TBC**
Range (μg/g)	1.3–7.6	4.9–26.7	1.0–7.0	0.1–1.2	1.1–6.1
Average (μg/g)	3.8 ± 1.3	14.7 ± 4.9	3.8 ± 1.3	0.5 ± 0.2	3.7 ± 1.2
*p* Genotype	***	***	***	***	***
*p* Maturity stages	***	***	***	***	***
*p* Processing methods	***	***	***	***	***
*p* Genotype * Maturity stages	NS	NS	NS	NS	NS
*p* Genotype * Processing methods	NS	NS	NS	NS	NS
*p* Maturity stages * Processing methods	***	***	***	***	***

Values are the results (average ± standard deviation) of triplicate determinations. ***p < 0.001; NS, non-significant (p > 0.05); ZXT, zeaxanthin; BCX, β-cryptoxanthin; AC, α-carotene; TBC, total β-carotene; SC_50_, concentration of maize extract that scavenged 50% of DPPH*.

Genotype, maturity stages and processing methods all had significant effects (*p* < 0.001) on the levels of all the carotenoids. The interaction of maturity stages and processing methods also had a significant effect (*p* < 0.001) on the levels of all the carotenoids.

### Phytate, Tannins, and Vitamin C Contents of Orange Maize Hybrids as Affected by Genotype, Maturity Stages, and Processing Methods

The concentrations of phytate, tannins, and vitamin C in the orange maize hybrids as affected by genotype, maturity stages, and processing methods are presented in Table 2. The concentration of phytate ranged from 0.7 to 3.0%, with an average value of 1.7%. Relative to the phytate level (1.71 g/kg), Horvatic and Balint (29) reported in fresh maize harvested at 57 days after planting, but the average level of phytate quantified in this present study is higher than that reported by them. Although phytate reduces the bioavailability of divalent metal ions, including zinc, iron and calcium in the gastrointestinal tract by chelating them (30), it has some health benefits. Its abilities to inhibit the development of kidney stone (31) and cancer (32) have been reported.

**Table 2:** Phytate, tannin, and vitamin C levels of orange maize hybrid as affected by genotype, maturity stages, and processing methods.

**Summary**	**Phytate (%)**	**Tannins (%)**	**Vit C (mg/100 g)**
Range	0.7–3.0	0.0–3.3	21.2–62.3
Average	1.7 ± 0.62	1.4 ± 0.8	34.8 ± 8.7
*p* Genotype	NS	NS	*
*p* Maturity stages	***	***	***
*p* Processing methods	***	***	***
*p* Genotype * Maturity stages	***	NS	NS
*p* Genotype * Processing methods	NS	NS	NS
*p* Maturity stages * Processing methods	***	***	***

Values are the results (average ± standard deviation) of triplicate determinations. *p < 0.05; ***p < 0.001; NS, non-significant (p > 0.05).

The level of tannins in the orange maize ranged from 0.0 to 3.3%, with an average of 1.4%. Relative to the range of tannins content observed in this present study, a range of 0.56–4.08% was reported for open-pollinated varieties of orange maize harvested at 20, 27, and 34 days after pollination, and subjected to boiling with and without husks (1). Adom and Liu (33) also reported that maize, with a total phenolics content of 15.55 ± 0.60 μmol/g of grain, was richer in total phenolics than some other commonly consumed cereals, including wheat (5.56 ± 0.17 μmol/g of grain), oats (6.53 ± 0.19 μmol/g of grain), and rice (7.99 ± 0.39 μmol/g of grain). Tannins and other phenolic compounds are well-known for their antioxidant activity, which they exhibit through various mechanisms, including scavenging of free radicals, chelating of metal ions, and inhibition of lipid peroxidation (34). In addition to their antioxidant activity, they possess other health benefits, including antidiabetic, anti-hyperuricemic, and anti-hypertensive activities (35, 36).

The vitamin C content of the orange maize hybrids was in the range of 21.2–62.3 mg/100 g, with a mean value of 34.8 mg/100 g. This agrees with the range of vitamin C (20.0–61.9 mg/100 g), Alamu et al. (1) reported in open-pollinated orange maize varieties harvested at 20, 27, and 34 days after pollination, and subjected to boiling with and without husks. Vitamin C is recognized as a major and powerful naturally occurring nutrient and antioxidant in humans' daily diet (37, 38).

Maturity stages and processing methods had significant effects (*p* < 0.001) on the levels of phytate, tannins and vitamin C. However, the genotypic effect was significant (*p* < 0.05) only on vitamin C content. The combined effects of genotype and maturity stages were significant (*p* < 0.001) on the level of phytate only. In contrast, the combined effects of maturity stages and processing methods were significant on the levels of phytate, tannins, and vitamin C.

### DPPH^*^ Scavenging Ability of Orange Maize Hybrids as Affected by Genotype, Maturity Stages, and Processing Methods

The free radical-scavenging activity of the orange maize hybrids, as determined using DPPH^*^ assay, is presented in Table 3. The DPPH^*^ SC_50_ (concentration of extract that scavenged 50% of DPPH^*^) of the maize ranged from 15.4 to 40.2 mg/mL, with an average value of 25.1 mg/mL, across the genotypes, maturity stages and processing methods. Yellow and white maize varieties had earlier been reported to scavenge DPPH^*^ (39), although the value was not expressed in SC_50_ terms. However, a comparison of the DPPH^*^ SC_50_ (0.035 mg/mL; data not presented in Table 3) of vitamin C, a reference antioxidant, and the range of orange maize DPPH^*^ SC_50_ (15.4–40.2 mg/mL) indicates that vitamin C had much stronger DPPH^*^ scavenging ability than the orange maize hybrids, since a lower DPPH^*^ SC_50_ is indicative of a stronger DPPH^*^ scavenging ability (36). The ability of the orange maize hybrids in this study to scavenge DPPH^*^ suggests that they may help in preventing the production of free radicals and/or mopping them up in the cell when consumed. Also, it may attenuate the oxidative degradation of some nutrients that are highly prone to oxidative spoilage in the orange maize hybrids, including unsaturated fatty acids and vitamins (11).

**Table 3:** DPPH^*^ SC_50_ of orange maize hybrids as affected by genotype, maturity stages, and processing methods.

**Summary**	DPPH* SC_**50**_ (mg/mL)
Range	15.4–40.2
Average	25.1 ± 5.07
*p* Genotype	***
*p* Maturity stages	NS
*p* Processing methods	***
*p* Genotype * Maturity stages	NS
*p* Genotype * Processing methods	NS
*p* Maturity stages * Processing methods	***

Values are the results (average ± standard deviation) of triplicate determinations. ***p < 0.001; NS, non-significant (p > 0.05); SC_50_, concentration of maize extract that scavenged 50% of DPPH*.

Genotype and processing methods had significant effects (*p* < 0.001) on the DPPH^*^ scavenging ability of the orange maize hybrids. Similarly, the interaction of maturity stages and processing methods also had a significant effect (*p* < 0.001) on the DPPH^*^ scavenging ability of the orange maize hybrids.

### Effect of Genotype on Bioactive Constituents (Carotenoids, Phytate, Tannins, and Vitamin C) and DPPH^*^ Scavenging Ability of Orange Maize Hybrids

Generally, genotype had a significant effect on the levels of lutein (*p* < 0.01), zeaxanthin (*p* < 0.001), β-cryptoxanthin (*p* < 0.001), α-carotene (*p* < 0.001), and total β-carotene (*p* < 0.001) in the orange maize hybrids (Table 4). This is consistent with the findings of some earlier studies that concluded that the levels of carotenoids in orange maize were genotype-dependent (1, 21). The levels of β-cryptoxanthin, α-carotene and total β-carotene were consistently and significantly higher (*p* < 0.001) in genotypes 5 and 7 than in genotype 4. The lutein levels of genotypes 4 and 5 were significantly higher (*p* < 0.01) than that of genotype 7. On the other hand, the zeaxanthin levels of genotypes 4 and 7 were significantly higher (*p* < 0.001) than that of genotype 5. Previous studies reported that genotypic variation in provitamin A carotenoids in maize was useful in the conventional breeding of provitamin A biofortified maize (21, 40). No significant difference (*p* > 0.05) was observed among the genotypes in terms of their phytate and tannins levels. Genotype 4 had the highest level of vitamin C, although it was comparable (*p* > 0.05) with that of genotype 5.

**Table 4:** LS means of bioactive constituents and DPPH^*^ SC_50_ of orange maize hybrids as affected by genotype.

**Genotype**	**Lutein (μg/g)**	**ZXT (μg/g)**	**BCX (μg/g)**	**AC (μg/g)**	**TBC (μg/g)**	**Phytate (%)**	**Tannins (%)**	**Vit C (mg/100 g)**	DPPH* SC_**50**_ (mg/mL)
4	4.0^a^	16.3^a^	3.3^b^	0.4^b^	3.2^b^	1.8^a^	1.4^a^	36.3^a^	23.7^b^
5	4.1^a^	11.6^b^	4.0^a^	0.5^a^	3.8^a^	1.7^a^	1.4^a^	35.0^ab^	26.9^a^
7	3.4^b^	16.2^a^	4.1^a^	0.5^a^	4.0^a^	1.7^a^	1.5^a^	32.9^b^	24.7^b^
*p*-value	*p* = 0.0016	*p* < 0.0001	*p* = 0.0005	*p* = 0.0004	*p* = 0.0003	*p* = 0.5376	*p* = 0.6989	*p* = 0.0207	*p* < 0.0001

Mean values with different superscript letters along the same column vary significantly at p < 0.05 (Vit C), p < 0.01 (lutein), and p < 0.001 (ZXT, BCX, AC, TBC, and DPPH* SC_50_). ZXT, zeaxanthin; BCX, β-cryptoxanthin; AC, α-carotene; TBC, total β-carotene; SC_50_, concentration of maize extract that scavenged 50% of DPPH*.

The DPPH^*^ SC_50_ values of genotypes 4 (23.7 mg/mL) and 7 (24.7 mg/mL) were also comparable, but both were significantly lower (*p* < 0.001) than that of genotype 5 (26.9 mg/mL). Expectedly, genotype 4 with the highest level of vitamin C, had the least DPPH^*^ SC_50_ value (the strongest DPPH^*^ scavenging ability), suggesting that vitamin C may have contributed more to the DPPH^*^ scavenging ability of the orange maize hybrids than the other bioactive constituents. Thus, genotype 4 may have a better antioxidant property than genotypes 5 and 7. This is supported by the findings of Sytarová et al. (41) that vitamin C strongly contributed to antioxidant activity determined by DPPH^*^ scavenging assay.

### Effect of Maturity Stages on Bioactive Constituents and DPPH^*^ Scavenging Ability of Orange Maize Hybrids

The levels of all the carotenoids (except α-carotene) were significantly higher (*p* < 0.001) in the maize harvested at the 34th day than at the 20th and 27th day after pollination (Table 5). This suggests that the carotenogenic process in the orange maize hybrids may have peaked at the 34th day after pollination. This is consistent with a previous study on open-pollinated varieties of orange maize, in which the maximum levels of carotenoids were detected on 34 days after pollination, relative to 20 and 27 days after harvest (1).

**Table 5:** LS means of bioactive constituents and DPPH^*^ SC_50_ of orange maize hybrids as affected by maturity stages.

**Maturity stage**	**Lutein (μg/g)**	**ZXT (μg/g)**	**BCX (μg/g)**	**AC (μg/g)**	**TBC (μg/g)**	**Phytate (%)**	**Tannin (%)**	**Vit C (mg/100 g)**	DPPH* SC_**50**_ (mg/mL)
20D	3.4^b^	12.4^c^	3.1^b^	0.5^a^	2.9^c^	1.4^c^	1.4^b^	34.7^b^	24.9^a^
27D	3.3^b^	14.3^b^	3.6^b^	0.4^b^	3.6^b^	2.1^a^	1.1^c^	30.1^c^	25.9^a^
34D	4.7^a^	17.4^a^	4.6^a^	0.5^a^	4.5^a^	1.7^b^	1.8^a^	39.5^a^	24.5^a^
*p*-value	*p* < 0.0001	*p* < 0.0001	*p* < 0.0001	*p* < 0.0001	*p* < 0.0001	*p* < 0.0001	*p* < 0.0001	*p* < 0.0001	*p* = 0.1163

Mean values with different superscript letters along the same column vary significantly at p < 0.001. 20D, 20 days after pollination; 27D, 27 days after pollination; 34D, 34 days after pollination; ZXT, zeaxanthin; BCX, β-cryptoxanthin; AC, α-carotene; TBC, total β-carotene; SC_50_, concentration of maize extract that scavenged 50% of DPPH*.

The phytate, tannins and vitamin C levels also varied significantly (*p* < 0.001) across the three maturity stages of harvest. Whereas, phytate level was in the order of 27D > 34D > 20D, the levels of tannins and vitamin C were both in the order of 34D > 20D > 27D. Contrary to these trends, in open-pollinated orange maize varieties harvested at the same stages of maturity as in this present study, Alamu et al. (1) observed that phytate and tannins levels decreased with increasing maturity, while vitamin C level increased across the three stages of maturity of harvest. The result further revealed that the DPPH^*^ SC_50_ values of the orange maize hybrids were comparable (*p* > 0.05) across the three maturity stages of harvest, although maize harvested at the 34th day after pollination had the least value (the strongest DPPH^*^ scavenging ability). Hence, for optimum carotenoids (lutein, zeaxanthin, β-cryptoxanthin, and total β-carotene), tannins and vitamin C concentrations, harvesting orange maize hybrids at the 34th day after pollination may be preferable than at the 20th and 27th day after pollination.

### Effect of Processing Methods on the Bioactive Constituents and DPPH^*^ Scavenging Ability of Orange Maize Hybrids

The LS means of the bioactive constituents and DPPH^*^ SC_50_ of the orange maize hybrids due to the different processing methods are presented in Table 6. Relative to the fresh maize, hydrothermal processing with and without husks, and dry-heating with husks resulted in a significant increase (*p* < 0.001) in the levels of lutein, β-cryptoxanthin, α-carotene, and total β-carotene. Among the processing methods, only dry-heating with husks had a significant effect (*p* < 0.001) on the level of zeaxanthin, as its level (17.0 μg/g) was higher when compared with that of the fresh maize (13.3 μg/g). However, the levels of all the carotenoids in the fresh maize were comparable (*p* > 0.05) with those subjected to dry-heating without husks. The observed increase in the concentrations of the carotenoids (lutein, β-cryptoxanthin, α-carotene, and total β-carotene) in the orange maize hybrids due to hydrothermal processing is consistent with the report of Muzhingi et al. (42) that cooking of yellow maize by boiling at 100°C for 30 min led to an increase in the concentration of carotenoids. This increase may be attributed to enhanced extractability of carotenoids as a result of the breakdown of tissues of the maize grains. It is known that carotenoids are sequestered into protein complexes in plant tissues, and that cooking facilitates their releases (43, 44). These may also explain the reason for the observed significant increase in the concentrations of lutein, β-cryptoxanthin, α-carotene, and total β-carotene as a result of dry-heating with intact husks. Whereas, heat treatment led to loss of provitamin A carotenoids in orange maize products (45) and yellow dent inbred maize lines (46), the intact husks of the orange maize cobs in this study may have protected the carotenoids from exposure to direct heat, and consequently from their degradation. Thus, instead of causing their loss by degradation, oxidation, and isomerization (45), dry-heating with intact husks may have increased the ease of extraction of the carotenoids from the maize grain tissues. This may also account for the relatively increased level of zeaxanthin in the orange maize subjected to dry-heating with intact husks. However, the levels of all the carotenoids in the orange maize hybrids were comparable in the fresh maize and in those subjected to dry-heating without husks.

**Table 6:** LS means of bioactive constituents and DPPH^*^ SC_50_ of orange maize hybrids as affected by processing methods.

**Processing method**	**Lutein (μg/g)**	**ZXT (μg/g)**	**BCX (μg/g)**	**AC (μg/g)**	**TBC (μg/g)**	**Phytate (%)**	**Tannins (%)**	**Vit C (mg/100 g)**	DPPH* SC_**50**_ (mg/mL)
FR	3.2^b^	13.3^bc^	3.1^b^	0.3^b^	3.1^b^	1.8^ab^	1.5^bc^	39.2^a^	18.9^d^
HTH	4.4^a^	15.5^ab^	4.2^a^	0.6^a^	4.2^a^	1.7^b^	0.7^d^	34.3^b^	27.3^b^
HTWH	3.9^a^	14.9^abc^	4.0^a^	0.6^a^	3.8^a^	1.4^c^	1.1^cd^	27.1^c^	30.4^a^
DHH	4.4^a^	17.0^a^	4.6^a^	0.5^a^	4.2^a^	2.0^a^	1.7^ab^	32.5^b^	24.8^c^
DHWH	3.1^b^	12.8^c^	3.0^b^	0.4^b^	3.1^b^	1.9^a^	2.0^a^	40.7^a^	24.2^c^
*p*-value	*p* < 0.0001	*p* < 0.0001	*p* < 0.0001	*p* < 0.0001	*p* < 0.0001	*p* < 0.0001	*p* < 0.0001	*p* < 0.0001	*p* < 0.0001

*Mean values with different superscript letters along the same column vary significantly at p < 0.001*.

FR, fresh (unprocessed); HTH, hydrothermal with husks; HTWH, hydrothermal without husks; DHH, dry-heating with husks; DHWH, dry-heating without husks; ZXT, zeaxanthin; BCX, β-cryptoxanthin; AC, α-carotene; TBC, total β-carotene; SC_50_, concentration of maize extract that scavenged 50% of DPPH*.

The level of phytate in the orange maize hybrids decreased significantly (*p* < 0.001) due to hydrothermal processing without husks in relation to the fresh orange maize and other processing methods, which were all comparable (*p* > 0.05). Heat treatment was reported to reduce the level of phytate in white and yellow maize (39). The level of tannins in the orange maize hybrids decreased due to hydrothermal processing with and without husks when compared with the fresh maize. This decrease may have been precipitated by the oxidation and/or leaching of the tannins into water during the hydrothermal processing, as earlier reported for other phenolic compounds (47). On the other hand, dry-heating with and without husks led to an increase in the tannins level, relative to the fresh orange maize. Similar to this finding, Belviso et al. (48) reported an increase in the levels of phenolic compounds, including condensed tannins, in licuri fruits after roasting. The increase in tannins level of the orange maize after dry-heating may be due to enhanced extraction of phenolic compounds with increase of temperature (49). In addition, the possible reaction of protein and sugars in the orange maize during the dry-heating process may have led to the formation of Maillard reaction products that also have the ability to react with Folin–Ciocalteu reagent, as phenolic compounds do, giving a higher tannins value (48). However, Irondi et al. (5) reported a decrease in the levels of phenolic compounds of roasted red sorghum grains, which was attributed to thermal decomposition of the phenolic compounds.

Relative to the fresh orange maize, the level of vitamin C decreased significantly (*p* < 0.001) due to hydrothermal processing with and without husks, and dry-heating with husks. This decrease may be due to thermal degradation of vitamin C, as vitamin C is known to be highly sensitive to heat treatment (50). The DPPH^*^ SC_50_ of the orange maize hybrids significantly increased (*p* < 0.001) in the processed maize in comparison with the fresh maize. The DPPH^*^ SC_50_ values were in the following order: hydrothermal processing without husks > hydrothermal processing with husks > dry-heating with husks > dry-heating without husks > fresh. This trend inversely shows the increasing strengths of the DPPH^*^ scavenging ability of the orange maize hybrids. Thus, the fresh maize had the strongest ability, followed by maize subjected to dry-heating without husks, dry-heating with husks, hydrothermal processing with husks and hydrothermal processing without husks. Therefore, the ability of the orange maize hybrids to scavenge DPPH^*^ decreased due to hydrothermal and dry-heating treatments. This decrease may be due to the degradation of the antioxidant bioactive constituents, especially vitamin C. However, the abilities of the orange maize hybrids subjected to dry-heating (with and without husks) to scavenge DPPH^*^ were stronger than those subjected to hydrothermal processing, suggesting that dry-heating treatment may have resulted in the formation of Maillard reaction products that may have contributed to the free radical scavenging ability of the maize. This is supported by the finding of Irondi et al. (5) that roasting caused an increased in the free radical scavenging ability of sorghum grains, due to the formation of Millard reaction products.

### Correlations of the Bioactive Components and DPPH^*^ Scavenging Ability of the Orange Maize Hybrids

The correlations among the bioactive constituents and DPPH^*^ SC_50_ of the orange maize hybrids are presented in Table 7. Alpha-carotene was significantly correlated (*p* < 0.05) with phytate, lutein, β-cryptoxanthin, total β-carotene, and zeaxanthin (*r*: −0.24, 0.57, 0.69, 0.62, and 0.39, respectively); lutein was correlated with each of β-cryptoxanthin, total β-carotene, and zeaxanthin (*r*: 0.57, 0.58, and 0.52, respectively); β-cryptoxanthin was correlated with total β-carotene and zeaxanthin (*r*: 0.92 and 0.63, respectively); total β-carotene was correlated with zeaxanthin (*r*: 0.65); tannins level was correlated with vitamin C and zeaxanthin (*r*: 0.41 and 0.26, respectively). The positive correlations observed between lutein and β-cryptoxanthin, lutein and zeaxanthin, as well as β-cryptoxanthin and zeaxanthin agree with the reports of previous studies in provitamin A biofortified maize (21, 40). Furthermore, the positive correlation between β-cryptoxanthin and total β-carotene in this study is in line with the findings of Suwarno et al. (40), who also reported a strong and highly significant correlation between β-cryptoxanthin and β-carotene in normal yellow maize. However, Muzhingi et al. (21) reported that these two carotenoids had no significant correlation in provitamin A biofortified maize.

**Table 7:** Correlations among bioactive constituents and DPPH* SC_50_ of orange maize hybrids.

**Variables**	**Vit C (mg/100 g)**	DPPH* SC_**50**_ (mg/mL)	**AC (μg/g)**	**Lutein (μg/g)**	**BCX (μg/g)**	**TBC (μg/g)**	**ZXT (μg/g)**	**Tannins (%)**
Phytate (%)	−0.11	−0.11	−0.24*	−0.04	−0.05	0.06	−0.02	−0.01
Vit C (mg/100 g)		−0.38***	−0.10	0.10	−0.03	−0.03	0.05	0.41***
DPPH* SC_50_ (mg/mL)			0.36***	0.21	0.29**	0.27*	0.03	−0.17
AC (μg/g)				0.57***	0.69***	0.62***	0.39***	0.08
Lutein (μg/g)					0.57***	0.58***	0.52***	0.14
BCX (μg/g)						0.92***	0.63***	0.16
TBC (μg/g)							0.65***	0.12
ZXT (μg/g)								0.26*

*p < 0.05; **p < 0.01; ***p < 0.001; ZXT, zeaxanthin; BCX, β-cryptoxanthin; AC, α-carotene; TBC, total β-carotene; SC_50_, concentration of maize extract that scavenged 50% of DPPH*.

Positive correlations were observed between DPPH^*^ SC_50_ and all the carotenoids quantified in the orange maize hybrids. However, since a lower DPPH^*^ SC_50_ represents a stronger DPPH^*^ scavenging ability as earlier stated, the carotenoids present in the orange maize hybrids may not have contributed significantly to the observed DPPH^*^ scavenging potency of the orange maize hybrids. A similar finding was reported by Muzhingi et al. (21), who found no significant correlations between the total antioxidant power and the carotenoids in provitamin A biofortified maize. They suggested that the higher levels of β-carotene and βcryptoxanthin in the provitamin A maize flour were insufficient to cause a higher antioxidant activity in the provitamin A biofortified maize they analyzed.

Contrary to the positive correlations observed between DPPH^*^ SC_50_ and the carotenoids in the orange maize hybrids, a significant and negative weak correlation (*p* < 0.001; *r*: −0.38) was observed between DPPH^*^ SC_50_ and the vitamin C content of the orange maize hybrids. Phytate and tannin also had negative correlations with DPPH^*^ SC_50_ (*r*: −0.11 and −0.17, respectively), but these were not significant (*p* > 0.05). The significant and negative correlation observed between DPPH^*^ SC_50_ and the vitamin C level indicates that vitamin C may have contributed more to the DPPH^*^ scavenging activity of the orange maize hybrids than the other bioactive constituents, as earlier stated. The potency of vitamin C as an antioxidant compound has consistently been reported in different foods (37, 38). Taken together, the correlations among the bioactive constituents and the DPPH^*^ scavenging ability of the orange maize hybrids observed in this study may be useful to breeding programmes targeting to improve the antioxidant properties of orange maize hybrids.

## Conclusions

Genotype had significant effect on the levels of the carotenoids and vitamin C; while maturity stages, processing methods and their interaction had significant effects on the levels of carotenoids, phytate, tannins, and vitamin C in the orange maize hybrids. Genotype and processing methods also had significant effects on the DPPH^*^ SC_50_ of the orange maize. Genotype 4 had a better combination of antioxidant constituents and free radicals scavenging ability than genotypes 5 and 7. Orange maize hybrids harvested at 34 days after pollination contained higher concentrations of carotenoids, tannins and vitamin C, and stronger DPPH^*^ scavenging ability than those harvested at 20 and 27 days after pollination. Hydrothermal processing with and without husks led to increase in the concentrations of the carotenoids in the orange maize hybrids. Fresh orange maize hybrids had the strongest DPPH^*^ scavenging ability, followed by orange maize hybrids subjected to dry-heating without husks. Important associations that can provide insight into breeding manipulations to improve the levels of bioactive constituents and the DPPH^*^ scavenging ability of the orange maize hybrids were also observed in this study.

## Data Availability Statement

The raw data supporting the conclusions of this article will be made available by the authors, without undue reservation.

## Author Contributions

EA and BM-D designed the study. EA conducted the experiments. EA and EI analyzed and interpreted the datasets and prepared the manuscript. AM and BM-D supervised the study and edited the manuscript. All authors contributed to the article and approved the submitted version.

## Conflict of Interest

The authors declare that the research was conducted in the absence of any commercial or financial relationships that could be construed as a potential conflict of interest.
